# COVID-19 Reflections and Team-Based Entrustable Professional Activities for Bioevent Preparedness

**DOI:** 10.15694/mep.2020.000265.2

**Published:** 2021-09-03

**Authors:** Sivakumar Arunachalam, Jitendra Sharan

**Affiliations:** 1 International Medical University; 2 International Medical University; 3 All India Institute of Medical Sciences; 4 All India Institute of Medical Sciences

**Keywords:** COVID-19, coronavirus disease 2019, SARS-CoV-2, Pandemic, Bioevent, Entrustable professional activities

## Abstract

This article was migrated. The article was marked as recommended.

The coronavirus disease 2019 (COVID-19) outbreak has shaken the entire human race with its unprecedented health and economic outcomes. Researchers and clinicians are trying to get an insight into various aspects of the COVID-19 disease and related aspects, and to some extent, they have succeeded too, but a lot more is yet to be discovered and disclosed. However, it appears that the pandemic is going to stay for a longer duration with varied expressions. In these testing and challenging times when the entire world is battling the massive public health crises, the healthcare workers remain the most vulnerable targets. The current situation unleashes adoption of new structures and processes, but often gloomed with ethical dilemmas. The COVID-19 is not going to be the last major pandemic in the history of human civilization. Henceforth, the million-dollar questions we face now and forever are: How are we planning to prepare the workforce for an health crisis of this cataclysmic proportions? Can we reimagine a curricular framework that could address public health crises arising from epidemics and pandemics? What model could facilitate for advancement in crisis preparedness and management? Do we need an orchestrated team approach in managing and containing any public health crisis of global concern like the COVID-19? What about the entrustable professional activities for a team task? These pertinent questions do not have straight forward answers. It requires a great effort at various levels, but such a change in curricular framework appears to be the need of the hour.

## Introduction

The world is facing a new global threat, i.e., the COVID-19. At present, more than 188 countries and territories have been affected by the severe acute respiratory syndrome coronavirus 2 ( *SARS-CoV-2*) (Coronavirus Resource Centre - Johns Hopkins University and Medicine), leading the Director-General of the World Health Organization (WHO) to declare the condition as ‘Public Health Emergency of International Concern’ (World Health Organization). As of August 22, 2020, more than 22.96 million COVID-19 cases have been reported across the globe, with the resultant deaths more than seven hundred thousand (Coronavirus Resource Centre - Johns Hopkins University and Medicine). The rapid spread of COVID-19 has resulted in unprecedented health, economic, and humanitarian crises. The world was not ready to face such a grievous scenario, leading to profound chaos and uncertainty. The large-scale public health crisis makes the COVID-19 as one of the dreadful pandemics in the history of human civilization. Our past experiences with health-related issues have suggested that the COVID-19 is going to stay with us for a considerable time. Did we refuse to learn the lessons from the past? The million-dollar question that we must face is: How are we equipped to prepare the workforce for a health crisis of this cataclysmic proportions, now and in the near future? The front-line healthcare providers are facing the crimson fear and confusion, such as, “Am I going to get infected even after taking appropriate precautions”, “Am I putting the personal protective equipment (PPE) correctly and ensuring scene safety”, “What if I do something wrong”, and the questions continue. Such questions frequently challenge patient and personal safety, along with team safety.

## The healthcare workforce and moral dilemmas

The World Health Organization estimated that healthcare providers, especially doctors, nurses, paramedics and carers, were 21 to 32 times more likely to get an Ebola infection (when the Ebola outbreak happened 6 years ago) than the general population (Nuntraand Anucha, 2015). The COVID-19 situation is more complex than Ebola. Globally, healthcare providers succumb to unprecedented workload, fear and mental stress while dealing with high-octane situations like this. Lack of skilled expertise to manage a local or regional response to the pandemic is an impending caveat ( Phua *et al*., 2020). We witnessed some of the institutions with a competency-based approach to medical education in the United States and Europe graduated their medical students (who have met their graduation requirements) earlier than the scheduled time and join the workforce to tackle the crisis ( Harley-McKeown and Korn, 2020). Such responses, which are secondary to the COVID- 19 pandemic, could change the thought process and formalize the concept based on exposure and competency attainment rather than the time alone to decide when the students should graduate. However, the thought that if this ‘just in time’ workforce is ready to meet the demands of the pandemic or any biological event disaster is equivocal.

The conundrum resides with the recruitment of less experienced healthcare workers (e.g., newly qualified students or healthcare workers moving from their specialism) to the workforce in response to the pandemic ( Bielicki *et al*., 2020). Another concern that revolves around the newly graduated workforce is their lack of preparedness for these global disruptions and potential susceptibility to moral trauma and adverse health outcomes ( O’Byrne, Gavin and McNicholas, 2020). The model of healthcare unleashed new teams, new workflow, new PPE protocols and scene safety, and new triage systems ( Bielicki *et al*., 2020; Gallagher and Schleyer, 2020). However, the scene was coupled with a lot of ethical dilemmas associated with the prioritization of treatment and logistical challenges (availability and commissioning of ventilators and PPEs) to critically ill COVID-19 patients or the COVID-19 patients who arrive with no co-morbidities ( Fritz *et al*., 2020).

## Reimagining curricular structure and student preparedness

In the times when we are pondering on how the world can navigate the pandemic, as educators, we are crammed with inquisitive thoughts to explore further. We need an unyielding focus on ways to tackle and control pandemics and seek to set out an emergency response system to adept in a curricular structure and transformation. The revamping needs to be designed largely in the curriculums in medical institutions, nurse education, and physician assistants. Patel and Dahl-Grove in 2018 assessed the medical students’ preparedness for a community event disaster as part of an elective study module and reported that 70% of the students felt unprepared to manage the event before the module. However, after the module, only 11% felt incompetent in disaster management ( Patel and Dahl-Grove, 2018). To date, only a few universities have incorporated disaster medicine or bioevent preparedness in their curriculum as an elective or extra-curricular program ( Smith *et al*., 2012; Kommor, Hodge and Ciottone, 2019). In a web-based survey of Italian undergraduate medical students, it was noted that the majority (91%) favored disaster medicine in their core curriculum and felt the importance of such modules to their career prospects ( Ragazzoni *et al*., 2013).

The COVID-19 is not going to be the last major pandemic in the history of human civilization. The residents and future doctors need to be leveled and calibrated to meet the demands of the epidemics and pandemics. One of the ways to adopt a core process in preparedness would include setting out educational objectives that engage students with approaches to handle end-of-life care in unprecedented situations, management of precarious situations with scarce resources (logistical challenges), consistent modeling of professionalism and leadership, and caring for patients who are non-compliant and quarantined. Certainly, outbreaks such as the COVID-19 will prompt us to reimagine our practices and prescriptions in medical education and healthcare functioning. A recent systematic review on student disaster training programs recommended a specific training program for medical students to improve preparedness, knowledge and skills in bioevent and disaster management ( Ashcroft *et al*., 2020).

Thus, can we reimagine a curricular framework that could address public health crises arising from epidemics and pandemics? What model could facilitate advancement in crisis preparedness and management? How do we establish a curricular structure that could integrate public health safety domains and translate the competencies of bioevent or pandemic management for a positive response? Do we need an orchestrated team approach in managing and containing any public health crisis of global concern? How about entrustable professional activities (EPAs) for a team task? These pertinent questions do not have straight forward answers. It requires a great effort at various levels, but such a change in curricular framework appears to be the need of the hour. Incorporating the changes in the education framework to address the public health crises of this magnitude can possibly keep the healthcare providers a step ahead.

## EPAs and a quest for special-operations health workforce federation model

EPAs-based curriculum in medical education is an innovative approach to educate the 21 ^st^ century millennials ( Ten Cate *et al*., 2015). EPAs are about responsibilities or tasks we as health care professionals have to do in patient care in our day-to-day practice. Thus, EPA based curriculum systematically prepares the students for safe, professional practice. It is about giving them the confidence to implement professional activity with continuous training. EPAs are gaining momentum in medical and paramedical education as a way of translating individual competencies into measurable units of clinical practice. The integration of domains of competence in a professional activity is the hallmark of the EPA concept ( Carraccio *et al*., 2017). The professional activities require specific knowledge, skills and attitudes and the student must be trained or learned the concepts and skills before performing the tasks. They should be observable and measurable in both process and outcome. The activities are executable within a given time frame and exist in a continuum. With every patient encounter, we use some of the competencies more than the others and integrate them into patient care in a holistic sense. The EPA concept is about giving confidence and trust to implement the activity, and it requires both theoretical and practical skill development with continuous training. In order for confidence to be rendered, the student must, therefore, be confident and have carried out such activity repeatedly without the supervisor having to intervene. The decision to trust a student with the critical responsibility to care for the patient is fundamental to clinical training.

Can we think of a platform where health advocacy (with clinical, civic, and humanitarian attributes) and collaboration could be reimagined to nurture the emerging public health crisis? Can we establish a special-operations health workforce federation, like military ‘special-operations forces’? The special-operations health workforce federation would serve as an elite system to address larger health concerns around the globe. The level of understanding and contemplation requires a guarded approach and team task.

## Team-based EPAs for bioevent preparedness – a cue for curriculum developers

COVID-19 pandemic has highlighted a number of vulnerabilities, including our lack of reinforcements, poor team connectedness and increased levels of anxiety and depression when attending to COVID-19 patients ( Beneria *et al.*, 2020). In the EPA-based curricular framework, entrustment decisions are made for a student based on various factors, including student functioning, supervisor willingness, the complexity of the task, dynamics of supervision, etc. The transfer of responsibility is generally contextualized in the same clinical setting. Moving forward with the idea and the concept of entrusting a team, the task can be judged and entrusted in different clinical contexts and situations accompanied by a regular sequence of training and exercises. Though there is no explicit ‘team’ entrustment in the clinical practice, few literature support team performances during simulation exercises and clinical practice focusing on the functioning of the total team as opposed to the performance of individuals within the team ( Fransen *et al.*, 2012; Miller *et al.*, 2012; Lerner, Magrane and Friedman, 2009). The core medical principles, leadership and professionalism, altruism, quality, and safety could be the core teachable attributes for the team. This may very well be the description of the EPA that could be designed for the ‘team task’ approach in entrustment. The team-based professional activities would encompass many activities but not limited to assessing the clinical signs in all spectrum of bioevent related ailments in the population, proper use personal protective equipment to reduce the risk of infectious exposures, familiarity with common diagnostic tests, prioritization of treatment, early management tactics in suspected cases, knowledge and skill acquisition in advanced airway management techniques or any emergency care tactics, initiation of ventilator management, management of complications associated with the therapeutics. Would team-based EPAs for bioevent preparedness make a difference in managing public health crises arising from the next epidemic or pandemic? Can the special-operations health workforce federation model become a reality to combat pandemics or any bioevent?

## Take Home Messages

• COVID-19 poses a major public health crisis with tremendous disruption to health and economy, along with the well-being of the global population.
• The front-line healthcare workers are the most vulnerable targets and the lack of preparedness of newly graduated workforce is a potential concern with serious implications to personal safety, patient safety and team safety.
• We need an unyielding focus on ways to tackle and control pandemics/bioevents with an emergency response system to adept in a curricular structure and transformation.
• Team-based Entrustable Professional Activities (EPAs) aligning with a conceptual model of special-operations health workforce federation could be a promising venture.

## Notes On Contributors

**Sivakumar Arunachalam** is a clinical orthodontist, educator and researcher in dentistry and Head of Children and Community Oral Health Division at School of Dentistry, International Medical Univerisity, Malaysia. He serves as chair for the EPA taskforce and a member of curriculum review board at School of Dentistry. ORCID ID: https://orcid.org/0000-0001-5300-5102

**Jitendra Sharan** is an academic and researcher in the area of biomaterials at All India Institute of Medical Sciences, Bhubaneswar, India. He serves as a member of the curriculum development committee at the institute. He has published widely in international and national journals, including assessment related textbooks. He is currently working with frontline health workforce team to combat COVID-19. ORCID ID: https://orcid.org/0000-0002-9788-8901
