# Supplementary material for: COVID-19 Reflections and Team-Based Entrustable Professional Activities for Bioevent Preparedness
Source: MedEdPublish (2016). 2021 Sep 3;9:265. Originally published 2020 Nov 27. [Version 2] doi: 10.15694/mep.2020.000265.2 (PMC10697441; doi:10.15694/mep.2020.000265.2)
Supplement: Peer review history

# Reviewer report: Reviewer response for version 2

**DOI:** 10.21956/mep.20285.r31497

**Published:** 2021-10-05

**Authors:** Ramon Esperon-Hernandez

**Affiliations:** 1 University of Yucatan

This review has been migrated. The reviewer awarded 4 stars out of 5 A relevant contribution taking into account that COVID-19 has not yet ended with more than 4.5 million deaths so far (CRC-JHUM).The questions are valid and the recommendations useful. Congrats.

Reviewer Expertise:

NA

No decision status is available

# Reviewer report: Reviewer response for version 2

**DOI:** 10.21956/mep.20285.r31496

**Published:** 2021-09-10

**Authors:** Ken Masters

**Affiliations:** 1 Sultan Qaboos University

This review has been migrated. The reviewer awarded 4 stars out of 5 The authors have addressed my concerns expressed about Version 1 of their paper, and have produced an opinion piece that is a valuable contribution to the topic. Thank you.

Reviewer Expertise:

NA

No decision status is available

# Reviewer report: Reviewer response for version 1

**DOI:** 10.21956/mep.19013.r27284

**Published:** 2021-02-16

**Authors:** Ken Masters

**Affiliations:** 1 Sultan Qaboos University

This review has been migrated. The reviewer awarded 3 stars out of 5 An interesting opinion piece on COVID-19 and team-based EPAs for bioevent preparedness.The strengths of the paper lie in the acknowledgment that there is a need for team-based EPAs in dealing with the COVID-19 pandemic and pandemics of the future. It is certainly evident that these do not currently exist. The context of this need, however, is not entirely clear: • Is their non-existence caused by the fact that there are no team-based EPAs in medical education AT ALL? Or • Is their non-existence caused by the fact that they DO exist in medical education, but just not for this specific type of event?If the former, then the paper would be strengthened by pointing out that COVID-19 has highlighted a weakness in current EPAs in general, as good teamwork is required in most areas of medical practice. If the latter, then it would be useful if the authors could draw on other team-based EPAs and lessons learned from them, to help formulate the argument for the need, and to give a clearer picture of the possible process ahead.Two other issues that I would like to see addressed:• A minor quibble: it’s probably not necessary to describe Heraclitus, as “a Greek philosopher”. In addition, given that sayings are so frequently erroneously attributed to great thinkers, it would be useful if the authors could supply a proper citation for that quotation.• There a several times when the spacing between words and sentences appears to be arbitrary. In most cases, this is merely an irritation; in other cases, it may alter the meaning. (e.g. in “tasks we as health care professionals have to do inpatient care in our day-to-day practice” it is not clear whether “inpatient” should be “in patient” or not). It would be useful if the authors could go through the piece and correct these errors.So, a useful piece – I look forward to Version 2 in which these issues are addressed.

Reviewer Expertise:

NA

No decision status is available

# Reviewer report: Reviewer response for version 1

**DOI:** 10.21956/mep.19013.r27285

**Published:** 2020-12-27

**Authors:** Olle ten Cate

**Affiliations:** 1 UMC Utrecht

This review has been migrated. The reviewer awarded 4 stars out of 5 What I like is how COVID-19 makes us rethink EPAs as units of professional practice not linked to one educational program only

Reviewer Expertise:

NA

No decision status is available
